# Remote symptom monitoring integrated into electronic health records: A systematic review

**DOI:** 10.1093/jamia/ocaa177

**Published:** 2020-09-23

**Authors:** Julie Gandrup, Syed Mustafa Ali, John McBeth, Sabine N van der Veer, William G Dixon

**Affiliations:** o1 Centre for Epidemiology Versus Arthritis, Division of Musculoskeletal and Dermatological Sciences, School of Biological Sciences, University of Manchester, Manchester, UK; o2 NIHR Greater Manchester Biomedical Research Centre, Manchester Academic Health Science Centre, University of Manchester, Manchester, UK; o3 Centre for Health Informatics, Division of Informatics, Imaging and Data Sciences, School of Health Sciences, Faculty of Biology, Medicine and Health, Manchester Academic Health Science Centre, University of Manchester, Manchester, UK; o4 Rheumatology Department, Salford Royal NHS Foundation Trust, Salford, UK

**Keywords:** remote monitoring, electronic health record, long-term conditions, digital health, mobile health, patient-generated health data

## Abstract

**Objective:**

People with long-term conditions require serial clinical assessments. Digital patient-reported symptoms collected between visits can inform these, especially if integrated into electronic health records (EHRs) and clinical workflows. This systematic review identified and summarized EHR-integrated systems to remotely collect patient-reported symptoms and examined their anticipated and realized benefits in long-term conditions.

**Materials and Methods:**

We searched Medline, Web of Science, and Embase. Inclusion criteria were symptom reporting systems in adults with long-term conditions; data integrated into the EHR; data collection outside of clinic; data used in clinical care. We synthesized data thematically. Benefits were assessed against a list of outcome indicators. We critically appraised studies using the Mixed Methods Appraisal Tool.

**Results:**

We included 12 studies representing 10 systems. Seven were in oncology. Systems were technically and functionally heterogeneous, with the majority being fully integrated (data viewable in the EHR). Half of the systems enabled regular symptom tracking between visits. We identified 3 symptom report-guided clinical workflows: Consultation-only (data used during consultation, n = 5), alert-based (real-time alerts for providers, n = 4) and patient-initiated visits (n = 1). Few author-described anticipated benefits, primarily to improve communication and resultant health outcomes, were realized based on the study results, and were only supported by evidence from early-stage qualitative studies. Studies were primarily feasibility and pilot studies of acceptable quality.

**Discussion and Conclusions:**

EHR-integrated remote symptom monitoring is possible, but there are few published efforts to inform development of these systems. Currently there is limited evidence that this improves care and outcomes, warranting future robust, quantitative studies of efficacy and effectiveness.

## INTRODUCTION

Nearly 1 in 4 adults across Europe and almost 1 out of 2 adults in the US are living with a long-term condition (LTC), and globally, LTCs are among the leading causes of years lived with disability.^1–3^ LTCs often require continuous management of care and life-long medication use, and the majority of health care spending in the developed world is in LTCs.^4^ As health care systems experience an increasing demand for services, there is a growing need to find innovative approaches to the provision and delivery of care to aid clinical and self-management of people living with an LTC.

At the same time, digital technologies are becoming increasingly pervasive, providing unique opportunities to collect health data directly from patients that can aid clinical decision-making and make care more patient-centric. Key features of patient-generated health data (PGHD) are: 1) the patient, not the health care provider, captures the data; 2) the data are obtained outside of clinical settings; and, therefore, 3) the data can be collected longitudinally and with high frequency.^5^ PGHD may include not only clinical data (such as home-based blood glucose measurements), but also other patient-reported aspects of health, such as symptoms, medical history, physical activity, and more. Some of these would be considered patient-reported outcomes (PROs). For the purpose of this review, we will focus exclusively on patient-reported symptom data, acknowledging that there is an overlap with certain PROs.

Collecting patient-reported symptom data remotely prior to a consultation might change clinical workflows, making them more efficient by not requiring patients to fill out assessments in the waiting room or reporting symptoms within the limited time patients have with their clinician during the clinic consultation. PGHD could also give a much clearer and complete picture of life outside of the clinic with more continuous, longitudinal monitoring. Longitudinal data could be used to inform ongoing care management and provide important insights into a patient’s health and well-being.^6^ Integrating this important information real-time with the electronic health record (EHR) would facilitate a more systematic symptom review at the point of care and allow tracking of symptom severity over time alongside other clinical information.^7–9^ Logging onto separate systems is a recognized barrier for clinicians to adopting a new health IT system, highlighting the importance of better integration.^10^^,^^11^ Integration into EHRs have been an aspiration for more than a decade, but despite the suggested benefits and opportunities of integrating patient-reported symptom data from remote monitoring into EHRs and clinical practice in LTCs, the supporting evidence for this remains unclear.^10^

## OBJECTIVE

No comprehensive systematic reviews exist of published EHR-integrated systems that remotely collect self-reported symptoms for clinical decision-making. Our aim was therefore to map the landscape of EHR-integrated remote symptom monitoring systems in the field of LTCs. Specifically, the objectives were to 1) characterize state of the art systems, 2) describe their use in clinical settings, and 3) outline the anticipated and realized benefits.

## MATERIALS AND METHODS

We designed and reported the systematic review according to (Preferred Reporting Items for Systematic Reviews and Meta-Analyses) PRISMA guidance.^12^

### Search strategy

We searched 3 electronic literature databases—Embase, MEDLINE, and Web of Science—until November 11, 2019. We were not interested in purely technical or system development papers, so we did not search computer science databases. The search strategy, which was developed in consultation with an experienced research librarian, consisted of a combination of Medical Subject Headings (MeSH) and free-text keyword terms related to the following 3 concepts: 1) long-term conditions including cancer,^13^ 2) patient-generated health data, and 3) data capture systems.^14^ We initially developed the search strategy in MEDLINE (see Supplementary Material  Table 1) and then adapted to other databases.

**Table 1.** Overview of studies included in the systematic review

Reference, (year)	Country	Type of study	Disease subtype	Number of patients	Setting	**Patient demographics: Age** ^a^ **Gender** **Ethnicity**	Commercial tool, (name)
Cancer							
Graetz et al (2018)	USA	Randomized controlled feasibility trial	Breast	44	Medical breast cancer center	59.9 [34; 77] 100% female 25% non-white	Not reported
Snyder et al (2013)	USA	Single-arm prospective pilot study	Breast, prostate	52	Academic cancer center	58 [28–81] 72% female 18% non-white	No (PatientViewPoint)
Warrington et al (2019)	UK	Observational clinical field testing	Breast	12	Medical oncology breast service in a cancer center.	47.5 (10.3) [33; 73] 100% female Not reported	No (eRAPID)
Zylla et al (2019)	USA	Prospective feasibility study	Non-hematologic	80	Large, urban community cancer center	62 [26; 85] (median) 66% female 4% non-white	Yes (EPIC MyChart)
Garcia et al (2019)	USA	Clinical quality improvement initiative	Various subtypes	3521	Medical oncology clinic	57.2 (13.4) 68.1% female 16.7% non-white	Yes (EPIC MyChart)
Wagner et al (2015)	USA	Implementation study	Gynecologic	636	Gynecologic oncology clinic	55.1 (12.8) [21; 90] 100% female 12.9% non-white	Yes (EPIC MyChart)
Girgis et al (2017)	Australia	Mixed methods feasibility study	Most subtypes	35	Two public hospital cancer centers	62.2 (11.2) [39; 85] 69% female Not reported	No (PROMPT-Care)
Van Egdom et al (2019)	The Netherlands	Overview of development and implementation	Breast	239	Academic Breast Cancer Centre	Not reported Not reported Not reported	Not reported
Rheumatology							
Austin et al (2019)	UK	Feasibility and acceptability study	Rheumatoid arthritis	20	Rheumatology clinic at a large, academic hospital	[32; 84] 75% female Not reported	No (REMORA)
Neurology							
Schougaard et al (2019)	Denmark	Parallel 2-arm pragmatic randomized controlled trial	Epilepsy	593	Academic neurology department	45.8 (17.1) 45% female Not reported	No (AmbuFlex)
Multiple disease areas							
Biber et al (2018)	USA	Overview of implementation experiences	All ambulatory clinics. From primary care to sub-specialty surgical practices	200.000	Large academic health care system	Not reported Not reported Not reported	No (mEVAL)
Schougaard et al (2016)	Denmark	Overview of implementation experiences	9 groups (Heart disease, epilepsy, narcolepsy, RA, sleep apnoea, prostate + colorectal cancer, asthma, renal failure)	Not reported	15 outpatient clinics in 1 region	Not reported Not reported Not reported	No (AmbuFlex)

a Mean age in years (standard deviation) [range].

### Selecting relevant studies

Studies were considered relevant if they met all of the following criteria:

- Evaluated symptom reporting systems, using a definition adapted from Vegesna et al:^15^ “An ambulatory, noninvasive digital technology used to capture patient data in real time and transmit health information for assessment by a health professional.” This evaluation excluded studies focusing on systems exclusively for sensor, wearable, implant, or biometric data, as they have been reviewed elsewhere.^15–17^
- Included adult patients living with an LTC as the study population, following the World Health Organization’s definition.^13^
- Facilitated a direct integration of digital patient-reported symptoms into the EHR on a single sign-on basis for the clinician.^18^
- Collected the symptom data remotely (ie, outside of conventional clinical settings). This excluded data collected on a tablet or computer in the waiting room before a clinic visit.
- Reported on systems that were used to communicate symptoms between patient and health care provider in a clinical consultation, thereby potentially influencing clinical decision-making. This excluded self–management-only systems.

Studies on video consultations were excluded, as we believe they represent a separate, distinct branch of telehealth. As we wanted a comprehensive overview of relevant systems, we did not exclude studies based on study design, quality, or sample size.

Retrieved records were imported into Endnote and deduplicated. Two reviewers, JG and SMA, independently screened titles and abstracts against the predefined inclusion criteria. For studies considered potentially relevant, we retrieved the full papers and 1 reviewer (JG) identified those meeting the criteria for inclusion. As a quality audit, a second person (SMA) reviewed a 10% random sample of full text references to check for agreement. The review team met regularly to align interpretations, and at each stage of the review process, discrepancies were solved through consensus discussion. Reference lists of included studies were additionally screened manually, as were reference list of recent important work in the field known to the authors.

### Data extraction and synthesis

We developed a data extraction form on the basis of the Office of the National Coordinator (ONC) for Health Information Technology PGHD white paper which presents a framework for describing the context and use of PGHD.^5^ It includes 3 steps in data flow: Capture (creation and storage of health data by the patient); transfer (communication of captured data to health care designees); and review (health care designee receiving the data and using it for decision-making).

We pilot-tested the extraction forms among the authors. The final list included items on study characteristics, technical and functional system specifications, response rate (defined as the author-reported percent completed questionnaires from the total eligible), clinical use, and anticipated and reported benefits of integrated symptom monitoring. JG reviewed and extracted data from eligible studies. Additional information on the systems was sought for the studies that had been described in detail elsewhere, such as in technical system architecture publications or protocols.

For objective 3, we were interested in seeing what kinds of anticipated benefits the authors thought were most important and if they succeeded in realizing any of them by looking at which benefits they evaluated. We adopted the 10 outcome indicators proposed by Chen et al to guide our evaluation of anticipated and realized benefits of remote symptom monitoring.^6^ The indicators aimed to evaluate the impact of routinely collected PROs on patients, service providers, and organizations (Supplementary Material  Table 2). They were initially developed for an oncologic setting, but as the frameworks upon which the 10 indicators rely are not disease-specific, it makes them useful for evaluating impacts beyond oncology.

**Table 2.** Specifications of the 10 systems for integrated remote patient-reported symptom monitoring

System	Data capture tool	EHR integration status for data^a^	Patient authentication	Data flow described?	Well described data security measures^b^	Option for patient to provide additional information	Feedback of own data to patient
Cancer							
Graetz et al	Website	Full integration	Not reported	Yes	Not reported	Not reported	Not reported
Snyder et al	Website	Full integration	Unique system log-in	Not reported	Yes	Yes	Yes Graphics of symptoms over time
Warrington et al	Website	Full integration	Unique system log-in	Yes	Yes	Yes	Yes Graphics of symptoms over time or written format
Zylla et al	Patient portal	Full integration	Personal patient portal log-in	Not reported	Not reported	Not reported	Not reported
Garcia et al Wagner et al	Patient portal	Full integration	Personal patient portal log-in	Yes	No	Not reported	Not reported
Girgis et al	Website	Full integration	Personal health identification or medical record number + password	Yes	No	Not reported	Not reported
Van Egdom et al	Website	Full integration	Not reported	Not reported	No	Not reported	Not reported
Rheumatology							
Austin et al	Smartphone app	Full integration	Unique system log-in	Yes	No	Yes	Yes Graphics of symptoms over time
Neurology							
Schougaard et al	Website	Partial integration	Personal health identification or Medical record number + password	Not reported	Yes	Not reported	Yes Graphics of symptoms over time
Multiple diseases							
Biber et al	Website	Full integration	Personal link. No need for log-in.	Not reported	No	Not reported	No

a “Full” integration allows data to be viewed from within the EHR. “Partial” has data available for review via a link inside the EHR that transfers the viewer to a secure website.

b Described in further detail than simply stating “firewall.”

We mapped each stated benefit against this list of indicators to be able to count and compare anticipated and realized benefits. Here, a benefit was defined as a positive result or consequence of integrated symptom monitoring stated by the authors. We classified benefits either as “anticipated” (what the authors stated as possible benefits in the introduction of their publication) or “realized” (supported by the study findings). Evidence for realized benefits was further categorized as quantitative, qualitative (eg, through interviews) or both; in the latter case, we counted an outcome twice for that study.

No attempt was made to quantitatively synthesize the results.

### Methodological quality assessment

Two reviewers (JG and SMA) independently evaluated the quality of each study reporting on realized benefits with the Mixed Methods Appraisal Tool (MMAT), which allows concomitant appraisal of quantitative, qualitative, and mixed-methods studies.^19^ Where discrepancies appeared, consensus was reached through discussion.

## RESULTS

**Figure 1.**
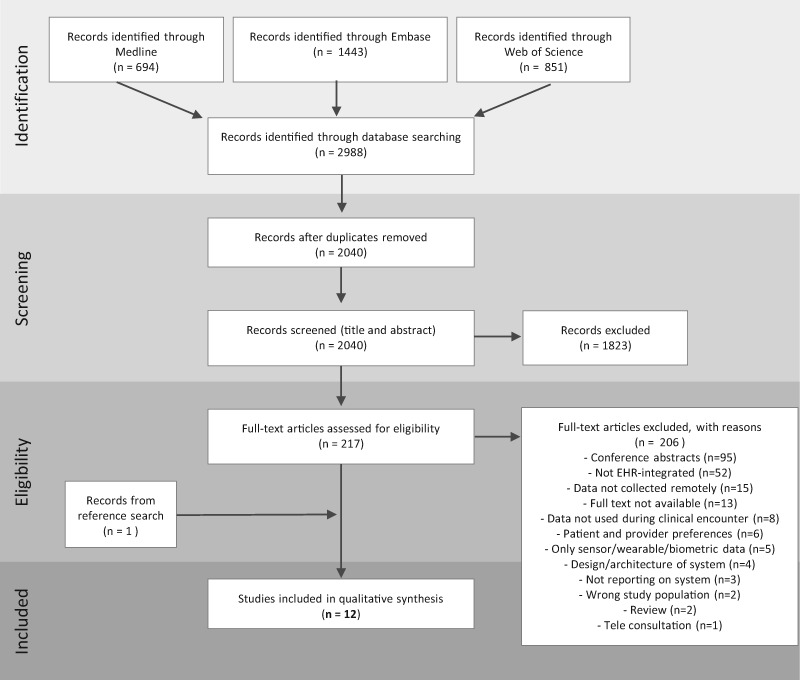
PRISMA flow diagram illustrating the systematic review process from electronic searching through to study inclusion.

Of 2040 articles identified through the search, 12 were selected for final inclusion, representing 10 unique systems. Figure 1 shows the PRISMA flow diagram depicting the review process.

All but 3 systems were used in oncology (Table 1).^20–22^ Half of the systems were in the United States, and only 3 systems were commercially available. Half of the systems were utilized for more than 1 disease subtype, such as tracking both breast and prostate cancer symptoms using PatientViewpoint^23^ or for 9 different diagnostic groups using AmbuFlex.^24^ The majority of studies were conducted in a single location.^20^^,^^21^^,^^23^^,^^25–30^

### System specifications

#### Data capture technologies

Table 2 shows that patient data capture technologies included 1 smartphone application available for Android phones^20^ and 2 online patient portals tethered to the EHR,^25^^,^^27^^,^^30^ but the majority of systems used websites that could be accessed from the patient’s home computer or any web-based device.^21–23^^,^^26^^,^^28^^,^^29^^,^^31^

#### EHR integration status

EHR integration was split into 2 categories based on where the data was viewed from: “full integration” and “partial integration.” Full EHR integration allowed data to be viewed and manipulated alongside other clinical data elements within the EHR. Nine out of 10 systems were fully integrated.^20^^,^^22^^,^^23^^,^^25^^,^^26^^,^^28–31^ The two online patient portals represented one type of fully integrated systems, and they were both EPIC MyChart portals. Registered patients could view portions of their medical record, add data to it, and exchange messages with physicians through a secure member website. One system represented partial integration, where data was available for review via a link inside the EHR that transferred the viewer to a secure website.^21^

There were different methods for displaying the data to the provider in the EHR. Garcia et al developed a system that displayed data as if they were lab results within the EHR.^25^ Austin et al’s smartphone app likewise had resultant PGHD immediately available in the EHR results section.^20^ Another way of displaying the data included a separate interface displaying symptoms graphs embedded in the EHR such as Warrington et al’s.^29^

#### Data flow and security

The flow of data from patient-facing technology to provider interface was described by half of the systems.^20^^,^^25^^,^^28^^,^^29^^,^^31^ Security measures were rarely described in detail. An example of well-described security measures came from Schougaard et al. They described how all data activities in the study were documented and stored in the WestChronic web system, where the system was located physically, and the specifications of the firewall. They described how backup was performed weekly and that all data transactions fulfilled conditions established by the Danish Data Protection Agency.^21^ In contrast, 5 systems only reported a “firewall,” and some did not describe security measures at all.^20^^,^^22^^,^^25^^,^^26^^,^^28^^,^^30^^,^^31^

#### Additional features

Graphical or written feedback of self-reported symptom data over time was available to patients in 4 systems.^20^^,^^21^^,^^23^^,^^29^ Three systems allowed patients to capture additional or contextual information in free text outside of the questions asked.^20^^,^^23^^,^^29^ Two systems provided self-management resources, including recommendations to manage milder symptoms,^29^ and e-mails with links to websites for managing symptoms exceeding predefined severity scores.^31^

### System usage

#### Frequency and purpose

As per Table 3, data collection frequency varied significantly, but overall fell into 2 groups: 1) longitudinal data collection at predefined intervals between visits, and 2) a single request before a scheduled clinic visit. For the longitudinal data group, patients were asked to report items with frequencies varying from daily to monthly.^20^^,^^23^^,^^28–30^ Additionally, 2 systems had the option for patients to report more frequently if desired. For systems with high reporting frequency, the duration of data collection per individual participant did not exceed 6 months, and, mostly it was less than 3 months. For some—and especially in cancer—the purpose was surveillance of patients undergoing toxic treatments; for others, it was to track fluctuating symptoms between follow-ups. Three systems also used the data as a basis for referrals to supportive care specialists, such as psychologists and nutritionists.^25^^,^^27^^,^^31^ The single request group reported symptoms just once in the lead-up to a scheduled outpatient visit, primarily with the purpose of replacing the typical waiting room or in-consultation assessments.^22^^,^^24–26^^,^^31^

**Table 3.** Type, duration, frequency and completeness of data collection by included systems for integrated remote patient-reported symptom monitoring

System	PGHD collected outcome instruments used	Number of items	Reporting frequency	Duration of data collection/study	Response rate, %^a^	Maximum data points per patient throughout study
Cancer						
Graetz et al	Physical symptoms Medication adherence	Not reported	Weekly + ad hoc	Individual: 6–8 weeks Study: 6 months	Not reported^b^	Unable to calculate
Snyder et al	Physical symptoms Psychological symptoms Quality of life Instrument: PROMIS	Not reported	Every 2 weeks	Individual: up to 6 months Study: 6 months	85% (190/224) overall. 71% by individual patient	Unable to calculate
Warrington et al	Physical symptoms Instrument: CTCAE	12 items	Weekly + ad hoc	Individual: app. 12 weeks Study: 3 months	63% (range 33%–92%)	144 items
Zylla et al	Physical symptoms Quality of life	23 items	Every 2 weeks	Individual: 12 weeks Study: app. 8 months	46% (125/271) were completed electronically. 66% (183/271) overall (range 58%–83%)	138 items
Garcia et al	Physical symptoms Psychological symptoms Supportive care needs Instrument: PROMIS CATs	App. 40 items	Before clinic visit	Individual: unknown Study: 2,5 years	51,6% (3521/6825) for any assessment	98 items
Wagner et al				Individual: unknown Study: 2 years, 3 months	36,8% for first assessment 34,5% for all assessments	104 items
Girgis et al	Physical symptoms Psychological symptoms Supportive care needs	47 items	Before clinic visit or Monthly	Individual: unknown Study: 3 months	77% (67/87) of assessments were completed^c^	141 items
Van Egdom et al	Physical symptoms Psychological symptoms Quality of life	Not reported	Before clinic visit	2 years evaluation (ongoing)	83.3% at baseline, 55.1% after 12 months overall	Unable to calculate
Rheumatology						
Austin et al	Physical symptoms Psychological symptoms	Daily: 9 Weekly: 11 Monthly: 23	Daily, weekly, monthly	Individual: 3 months Study: unknown	91% (range 78–95%)	1011 items
Neurology						
Schougaard et al	Physical symptoms Psychological symptoms Medication adherence Quality of life	48 items	Needs-based or Before clinic visit	Individual: 18 months Study: 24 months	Not applicable (Needs-based)	Unable to calculate
Multiple disease areas						
Biber et al	Physical symptoms Psychological symptoms Quality of life Instrument: PROMIS CATs	Not reported	Before clinic visit	Individual: unknown Study: 15 months (but ongoing effort)	47% overall. 17 %/47% at home	Unable to calculate
Schougaard et al	Physical symptoms Psychological symptoms Quality of life	Not reported	Before clinic visit	Unknown (ongoing)	81–98% across disciplines for initial assessment. 90–98% for follow-up	Unable to calculate

Abbreviations: CATs, computerized axial tomography scan; patient-generated health data; PROMIS, CTCAE, common terminology criteria for adverse events; PGHD,Patient-Reported Outcomes Measurement Information System.

a Response rate defined as percent completed questionnaires from total eligible. For highest frequency of reporting option within each system (eg, daily for Austin et al.).

b Used mean app use rate instead [Mean app use rate was 55%, defined as (number of reports/number of weeks enrolled)].

c Only shown overall including in-clinic completion and not specifically for home assessments.

#### Type and number of items collected

We identified 5 groups of collected patient-reported data: physical symptoms, psychological symptoms, quality of life, supportive care needs, and medication adherence.^32^ All systems included physical symptoms. Seven out of 12 references described reporting in 3 or more groups, most commonly a combination of physical and psychological symptoms and quality of life.^21–26^^,^^31^ Two systems used Patient-Reported Outcomes Measurement Information System computer adaptive tests.^22^^,^^25^

The maximum number of items requested per session ranged from 9 to 48 across systems. Generally, the systems that reported less often requested the highest number of items (> 40 items per reporting). However, the number of items requested was not available for 5 of the included systems.^22–24^^,^^26^^,^^28^

#### Response rate

Austin et al’s smartphone app had the highest response rate of 91% (range 78%–95%), despite asking patients to report on a daily basis.^20^ Similar rates were found across disciplines for Schougaard et al’s AmbuFlex system that asked to report before a visit (81%–98%).^24^ The lowest rates were found among the systems using patient portals (35%–52%), but, in contrast to the other systems, these were tested in naturalistic rather than more controlled settings.^25^^,^^27^^,^^30^ All 10 systems provided prompts to the patient when they were due to report.

### Clinical use

#### Workflow

We observed similarities in how the symptom data was integrated into clinic workflows, and synthesized them into 3 categories (Table 4). Five systems described a “consultation-only” workflow, which meant that the clinician viewed symptom data in the EHR just before or during the clinic consultation and inspected it with or without the patient to inform discussions and decision-making.^20^^,^^22^^,^^23^^,^^26^^,^^31^ An “alert-based” workflow included alerts to the clinical team when symptoms exceeded a predefined score (see below), but was otherwise similar to the “simple” workflow; this was described by 3 systems.^23^^,^^25^^,^^28^^,^^29^ Finally, 1 “on-demand” workflow meant that patients were sent questionnaires every 3, 6, or 12 months to guide their visit scheduling.^24^ Responses were given a green, yellow, or red color by a predefined automated algorithm. Green responses were handled automatically by the software. Yellow and red responses were shown on an alert list, where clinicians decided whether the patient needed a visit. A moderation to the “on-demand” workflow allowed the patients to indicate a need for contact by filling in questionnaires only when they felt they needed a visit.^21^

**Table 4.** Clinical use of integrated remote patient-reported symptom monitoring systems

System	Workflow	Alerts to care team	Results guide the frequency or format of consultations	Format of provider feedback	Provider training in use and interpretation
Cancer					
*Longitudinal monitoring between visits*					
Graetz et al	Alert-based	Yes to clinical team	Depends on action by medical team	Graphical depiction over time	Not reported
Snyder et al	Consultation-only	No	No	Graphical depiction over time	Yes
Warrington et al	Alert-based	Yes to clinical team	Depends on action by medical team	Plain-text table, highlighting with an asterisk	Yes
Zylla et al	Alert-based	Yes to clinical team	Depends on action by medical team	Graphical depiction over time	Not reported
*Single request before visits*					
Garcia et al and Wagner et al	Alert-based	Yes to clinical team + supportive care providers	No	Not reported	Not reported
Girgis et al	Consultation-only	No	No	Graphical depiction over time	Yes
Van Egdom et al	Consultation-only	No	No	Graphical depiction over time	Not reported
Rheumatology					
*Longitudinal monitoring between visits*					
Austin et al	Consultation-only	No	No	Graphical depiction of over time	Not reported
Neurology					
*Needs-based follow-up visits*					
Schougaard et al	On demand	Yes to clinical team	Yes	Graphical depiction over time	Not reported
Multiple diseases					
*Single request before visits*					
Biber et al	Consultation-only	No	No	Graphical depiction over time	Yes

Definitions: Alert-based, real-time alerts for providers when reporting severe symptoms; Consultation-only, data only used during consultation; On-demand, patient-initiated visits.

#### Alerts

After patients completed their questionnaires, 5 systems sent real-time alerts triggered by patient responses exceeding predefined thresholds primarily directed to staff.^21^^,^^25^^,^^28–30^ Alerts were either automated e-mails or EHR in-basket messages, and were most commonly set up to prompt follow-up by the treating clinician, nurse, or research coordinator. One oncology system additionally generated automatic referrals to nutritionists, social workers, and other supportive staff.^25^^,^^27^

### Anticipated and realized benefits

**Figure 2.**
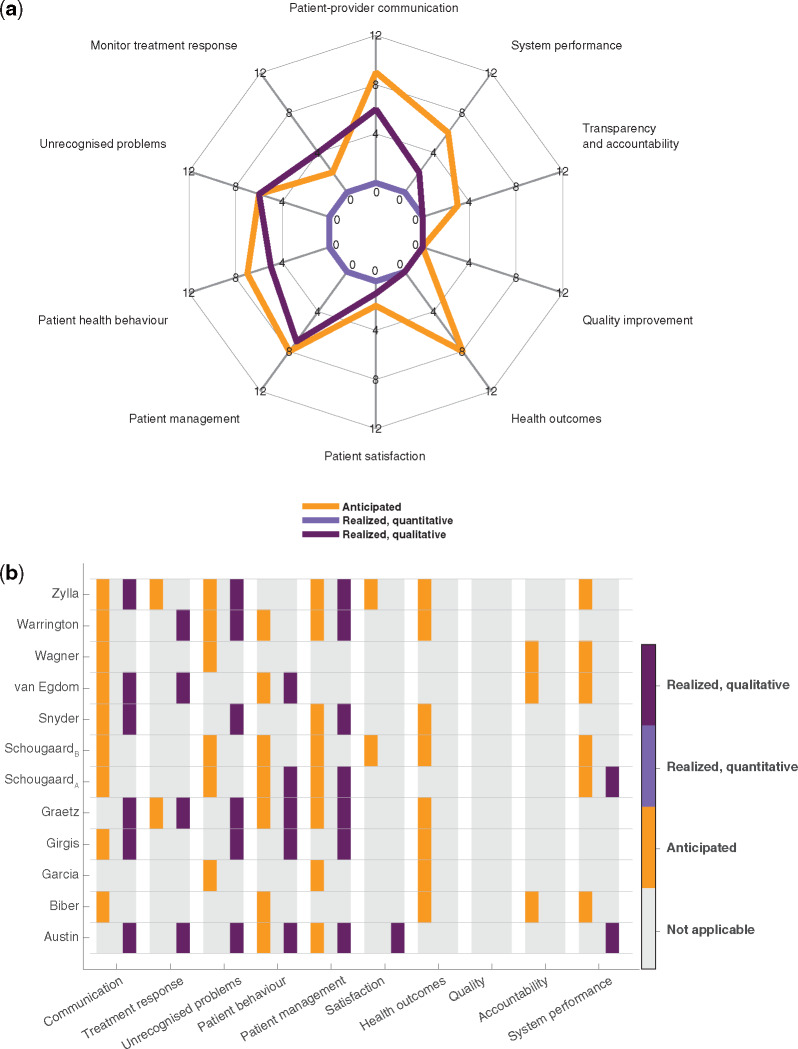
Summarized counts of anticipated and realized benefits showing that anticipated benefits outweigh realized benefits and that the latter are solely qualitative. (a) Spider plot illustrating summarized counts of benefits categorized after Chen et al’s 10 outcome indicators. Divided into anticipated (orange), realized quantitative (light purple), and realized qualitative (dark purple) benefits. (b) Heat map showing individual included references and their benefits in each of the categories: anticipated, realized quantitative, and realized qualitative benefits. Color convention as in (a).

From Figure 2, it is evident that there were several anticipated benefits to routine symptom reporting, but that few were actually realized. Improved health outcomes were particularly anticipated, but no study provided evidence for achieving these benefits. Evidence for the benefits that were realized was primarily of a qualitative nature. They involved better patient-provider communication, detection of unrecognized or hidden problems, changes to patient management, such as clinical management and decision-making, and changes to patient health behavior, including patient self-management and patient empowerment.

Two randomized controlled studies were the only studies that sought to provide quantitative evidence. However, neither of the studies compared integrated remote symptom monitoring to usual care without monitoring or to other types of symptom monitoring approaches; Schougaard et al compared patient-initiated to fixed interval PGHD-based follow-up,^21^ while Graetz et al compared symptom and medication adherence reporting with reminders to reporting without reminders.^28^ Therefore, no studies reported on the quantitative evidence of benefits that we were interested in for the purpose of this review.

### Quality assessment

Ten out of 12 studies were pilot, implementation, acceptability, or feasibility studies. Six studies reported on both qualitative and quantitative methods and were therefore classified as mixed methods. Results from the MMAT quality appraisal showed that most studies were of acceptable quality (see Supplementary Material  Table 3), though the qualitative domains generally showed higher quality than the quantitative. Most quantitative descriptive studies lacked a representative sample, while the two randomized trials both lacked blinding and suffered from high dropout rates, which lowered their quality. The mixed method studies appropriately used the design and integrated the data well, but none considered divergent qualitative and quantitative findings, which could indicate some outcome reporting bias.

Four studies were not appraised, as they did not report on realized benefits. These studies reported on experiences with implementation in larger health systems. Overall, they discussed challenges with system-wide implementation, what is essential for a successful process, and summarized metrics supporting the feasibility of such an approach.

## DISCUSSION

This systematic literature review of EHR-integrated remote symptom monitoring systems to support LTC management resulted in a heterogeneous list of 10 systems of which 7 were developed in oncology settings. Half of the systems requested a single symptom report ahead of a scheduled appointment while the other half allowed regular symptom tracking between visits. Systems moderated clinical workflows in 3 different ways: using data only during consultations, generating real-time alerts to providers, and scheduling outpatient visits. Of the anticipated key benefits, only a few benefits were realized and solely supported by qualitative evidence. Realized benefits included better patient–provider communication, detection of unrecognized or hidden problems, and changes to patient management.

The reported benefits should be viewed cautiously in light of aspects of study design. The majority of studies were early stage research, such as feasibility, pilot, and acceptability studies, and drawing conclusions on effectiveness should generally be avoided. Potential selection biases were present in a subgroup of studies where patients were identified by clinical staff or self-selected.^20^^,^^29^^,^^31^ The acceptability of these systems to other, perhaps less enthusiastic, participants, early and late adopters of technology, and different levels of digital literacy, is unknown. Most systems were implemented in a single setting, thereby limiting the generalizability of their results. Despite being 1 of the bigger concerns ,^33^ security measures were infrequently described. For the purpose of replication and providing blueprints for EHR integration moving forward, technical aspects need to be reported in more detail.

### Limitations

Although our search was comprehensive, it is possible we missed some systems. In particular, unpublished initiatives, remote symptom monitoring modules integrated into larger EHR systems, and systems that were used to collect data in the waiting room but may have had the capability to support symptom reporting from home.

The anticipated benefits summarized in this article included only those that the authors stated within the introduction section. It is possible that authors considered the anticipated benefits of remote monitoring to be wider but were not comprehensive in describing them.

### Other PGHD systems

Although out of scope for our review, PGHD systems focusing on aspects other than symptoms have been integrated in EHRs. Examples include diabetes and glucose measurements,^34^ hypertension and blood pressure measurements,^35^ and asthma and peak flow monitoring.^36^ Limitations shared among these efforts include low numbers of included patients, few engaged providers, and difficulties in displaying patient-reported data in a useful way within the EHR. Nonetheless, developing efficient ways to incorporate multiple types of PGHD in the EHR opens up a platform for capturing additional data types that further support the shift in clinical care models. However, problems of data integration are compounded by problems of visualization and making sense of large amounts of PGHD. At the moment, it is unclear how best to present PGHD to patients and clinicians in order to make the data meaningful in the clinical context. One solution to unlocking the value of PGHD while simultaneously avoiding information overload is visual analytics.^37^ Visualizing health data in a smarter and more interactive manner by leveraging visual analytics might aid the interpretation of complex health data, but more user-centered research is needed to better understand how this works in LTCs. There is, however, the necessary challenge of graph literacy in the general population if graphical data are to be used as a tool to support shared decision-making.^38^

Noah et al evaluated randomized controlled trials that assessed the effects of using noninvasive wearable biosensors for remote patient monitoring on clinical outcomes.^17^ They found that, while some remote monitoring interventions proved promising in changing clinical outcomes, there are still large gaps in the evidence base. Like us, they were limited by high heterogeneity and scarcity of high-quality studies, indicating that high-quality evaluations are warranted across the broader field of remote patient monitoring.

### Calls to action

Based on the findings presented in this review, we suggest 3 calls to action for harnessing the potential of integrated remote symptom monitoring: i) strengthening the quantitative evidence base, ii) accelerating work beyond oncology, and iii) improving interoperability. Below we outline each of these.

#### Strengthening the quantitative evidence base

The large number of pilot, implementation, and feasibility studies in our review demonstrate an emerging field. The next stride will be to quantitatively evaluate the effect of these systems in larger, more diverse populations. The National Institute for Health and Care Excellence has defined what good levels of evidence for digital health care technologies look like in the United Kingdom.^39^ Based on functions and potential user risks, technologies are stratified into evidence tiers. Symptom tracking functions that connect with a health care professional require Tier 3a evidence, the minimum standard being evidence from a high-quality observational or quasi-experimental study demonstrating impact on relevant outcomes, and should present comparative data. None of our included studies reached this level of evidence. Future studies should deliver this high-quality knowledge base.^40^

#### Accelerating work beyond oncology

Collecting PGHD remotely provides an opportunity for making consultations more efficient and patient-centric, while repeated collection could give a more complete picture of the patient and allows for continuous monitoring. The majority of our included studies were used within oncology, and 4 out of 5 systems that examined longitudinal monitoring between visits were used in cancer patients. Extrapolating these findings to other LTCs warrants caution, since oncology treatment regimens tend to be short-term instead of long-term, focus on monitoring side effects rather than symptoms related to the underlying condition, and patients might have different motivators to monitor symptoms during serious illness or end-of-life care. Recently, a randomized trial showed that monitoring chemotherapy side effects improved quality of life, acute hospital admissions, and survival.^41^ Despite not being integrated with the EHR, similar evidence from remote symptom monitoring on patient outcomes are rare across other LTC. Whether and how these results generalize from cancer to other LTCs is unknown, and accelerating work in fields outside of oncology is therefore highly encouraged.

#### Improving interoperability

Undeveloped interoperability standards were one of the challenges of PGHD integration laid out in the Office of the National Coordinator for Health Information Technology (ONC) report.^33^ Although not specifically addressed in any of the included papers, we found that each system developed their own technical infrastructure for integration. The emerging data exchange standard called Fast Healthcare Interoperability Resources (FHIR)^42^ and its latest extensions, SMART-on-FHIR and SMART Markers,^43^^,^^44^ are approaches developed to streamline and simplify EHR integration. Leveraging standards-based data exchange through interoperability could potentially both solve the interoperability challenge proposed by the ONC as well as ease EHR integration, making it an achievable goal for more health care systems.

## CONCLUSION

This systematic review shows that despite having been an aspiration for decades, there are few published studies to inform future development of EHR-integrated remote symptom monitoring systems for LTC care, but that integration is achievable. We found early indications from qualitative studies in support of integrated remote symptom reporting being beneficial, but these findings must be interpreted with caution. This implies we are still in the era of promise rather than realization when it comes to integrating patient-reported symptom data into the EHR. The next step will be for robust, quantitative studies to provide evidence of benefits—particularly beyond oncology.

## FUNDING

This work was supported by the Centre for Epidemiology Versus Arthritis (grant number 21755). The funder did not contribute to the concept, design, data collection and interpretation, or drafting of this manuscript.

## AUTHOR CONTRIBUTIONS

JG and SMA conducted the literature search and systematic review, and JG abstracted the data. JM, SNV, and WGD assisted in developing the research question and synthesizing the results. JG drafted the manuscript. All authors were involved in revising the manuscript critically, and all authors approved the final version to be submitted for publication.

The first and senior author (JG) had full access to all the data in the study and had final responsibility for the decision to submit for publication.

## SUPPLEMENTARY MATERIAL

Supplementary material is available at *Journal of the American Medical Informatics Association* online.

## Supplementary Material

ocaa177_Supplementary_Data
