# Supplementary material for: Remote symptom monitoring integrated into electronic health records: A systematic review
Source: J Am Med Inform Assoc. 2020 Sep 23;27(11):1752–63. doi: 10.1093/jamia/ocaa177 (PMC7671621; doi:10.1093/jamia/ocaa177)
Supplement: ocaa177_Supplementary_Data [file ocaa177_supplementary_data.pdf]

Supplementary material

Supplementary table 1. Final search query from MEDLINE via Ovid

Long-term conditions	Patient-generated health data	Data capture systems
1. Chronic Disease/<br>2. ((chronic* or persistent or long* term or ongoing) adj (disease* or disab* or ill* or condition* or health condition* or medical condition*)).mp.<br>3. Cardiovascular Diseases/<br>4. ("heart disease*" or "heart failure" or "myocardial ischemia" or "coronary disease *" or "coronary artery disease*" or "myocardial infarct*" or hypertension or "high blood pressure").mp.<br>5. Heart Failure/<br>6. Lung Diseases, Obstructive/<br>7. Pulmonary Disease, Chronic Obstructive/<br>8. ("obstructive lung disease*" or "obstructive pulmonary disease*" or copd or asthma or bronchitis).mp.<br>9. Cystic fibrosis/<br>10. cystic fibrosis.mp.<br>11. Stroke/<br>12. Stroke.mp.<br>13. (cerebrovascular or "brain isch?emia" or "cerebral infarc*" or "carotid artery disease*" or stroke or epilep* or seizure*).mp.<br>14. (Huntington* or Parkinson* or "amyotrophic lateral sclerosis" or "multiple sclerosis" or "motor neuron disease").mp.<br>15. Colonic Diseases, Functional/<br>16. Irritable Bowel Syndrome/<br>17. Irritable bowel syndrome.mp.<br>18. Musculoskeletal Diseases/<br>19. (arthritis or RA or osteoarthritis or rheumati* or fibromyalgia).mp.<br>20. Renal Insufficiency, Chronic/<br>21. ((renal or kidney) adj (failure* or insufficienc*)).mp.<br>22. Diabetes Mellitus/<br>23. Diabetes Mellitus, Type 1/<br>24. Diabetes Mellitus, Type 2/<br>25. (diabetes or diabetic*).mp.<br>26. Neoplasms/<br>27. (cancer* or oncolog* or neoplasm* or carcinom* or tumo?r* or malignan* or leuk?emia).mp.<br>28. Bipolar Disorder/<br>29. bipolar disorder.mp.<br>30. Schizophrenia/<br>31. schizophrenia.mp.<br>32. ((mental* or psychiatr* or psychological* or behavio*) adj (ill* or disorder* or disease* or distress* or disab* or problem* or health* or patient* or treatment)).mp.	33. Patient Generated Health Data/<br>34. (patient-generated health data or PGHD).mp.<br>35. patient-generated health information.mp.<br>36. patient-generated data.mp.<br>37. (patient-generated or person-generated or caregiver-generated or consumer-generated).mp.<br>38. Patient Reported Outcome Measures/<br>39. patient reported outcome*.mp.<br>40. patient reported outcome measure*.mp.	41. Remote patient monitoring.mp.<br>42. (digital adj2 (monitor* or track* or report* or record*)).mp.<br>43. (remote adj2 (monitor* or track* or report* or record*)).mp.<br>44. (electronic adj2 (monitor* or track* or report* or record*)).mp.<br>45. (tele adj2 (monitor* or track* or report* or record*)).mp.<br>46. (computer-based adj2 (monitor* or track* or report* or record*)).mp.<br>47. (smartphone adj2 (monitor* or track* or report* or record*)).mp.<br>48. (symptom adj2 (monitor* or track* or report* or record*)).mp.<br>49. Cell Phone/<br>50. Smartphone/<br>51. Smartphone.mp.<br>52. iPad.mp.<br>53. Mobile Applications/<br>54. Mobile application*.mp.<br>55. (mhealth or mobile health).mp.<br>56. Telemedicine/<br>57. telemedicine.mp.<br>58. digital health.mp.<br>59. Electronic Health Records/<br>60. (electronic health record* or electronic medical record* or electronic patient record*).mp.<br>61. Patient Portals/<br>62. Patient portal*.mp.<br>63. Health Records, Personal/<br>64. Personal health record.mp.<br>65. self track*.mp.<br>66. Health IT.mp.<br>67. Medical Informatics/<br>68. health information technology.mp.
69. 1 or 2 or 3 or 4 or 5 or 6 or 7 or 8 or 9 or 10 or 11 or 12 or 13 or 14 or 15 or 16 or 17 or 18 or 19 or 20 or 21 or 22 or 23 or 24 or 25 or 26 or 27 or 28 or 29 or 31 or 32<br>70. 33 or 34 or 35 or 36 or 37 or 38 or 39 or 40<br><br>71. 41 or 42 or 43 or 44 or 45 or 46 or 47 or 48 or 49 or 50 or 51 or 52 or 53 or 54 or 55 or 56 or 57 or 58 or 59 or 60 or 61 or 62 or 63 or 64 or 65 or 66 or 67 or 68<br>72. 69 and 70 and 71		

Supplementary table 2. Outcome indicators adapted from Chen et al., against which anticipated and realized benefits from the included studies were assessed.

Number	Outcome
1	Patient-provider communication
2	Monitor treatment response
3	Detect unrecognised problems
4	Changes to patient health behaviour
5	Changes to patient management
6	Improved patient satisfaction
7	Improved health outcomes
8	Strong and effective quality improvement
9	Increased transparency, accountability, public reporting
10	Better system performance (monitoring, planning, financing, evaluating, responding)

Chen J, Ou L, Hollis SJ. A systematic review of the impact of routine collection of patient reported outcome measures on patients, providers and health organisations in an oncologic setting. BMC Health Serv Res 2013;13:211. doi:10.1186/1472-6963-13-211

Supplementary table 3. Risk of bias for studies reporting on realized benefits based on criteria from the Mixed Methods Appraisal Tool (MMAT)

**Domain**	**Criterion**	Austin et al.	Biber et al.*	Garcia et al.*	Girgis et al.	Graetz et al.	Schou-gaard et al.*	Schou-gaard et al.	Snyder et al.	Van Egdom et al.	Wagner et al.*	Warring-ton et al.	Zylla et al.
Screening question	Clear research question	✓			✓	✓		✓	✓	✓		✓	✓
	Data adequate to address research questions	✓			✓	✓		✓	✓	✓		✓	✓
Qualitative	Appropriate qualitative approach	✓			✓				✓	✓		✓	
	Adequate data collection methods	✓			✓				✓	✓		✓	
	Findings adequately derived from data	✓			✓				?	?		✓	
	Interpretation substantiated by data	✓			✓				✓	?		✓	
	Coherence: data sources, collection, analysis and interpretation	✓			✓				✓	?		✓	
Quantitative descriptive	Relevance of sampling strategy	✓			?				✓	✓		✓	✓
	Representative sample	X			X				?	?		X	X
	Appropriate measures	✓			✓				✓	✓		✓	✓
	Low risk of non-response bias	?			?				?	?		?	?
	Appropriate statistical analysis	✓			✓				✓	?		✓	✓
Randomized controlled trial	Appropriate randomization					✓		✓					
	Comparable groups at baseline					✓		✓					
	Complete outcome data					✓		✓					
	Blinding					X		X					
	Low drop-out rate					X		X					
Mixed methods	Appropriateness of mixed-methods design	✓			✓				✓	✓		✓	

	Effective integration of quant. and qual. data	✓			✓				✓	✓		✓	
	Adequate outcome interpretation	✓			?				✓	✓		✓	
	Considerations of divergent quantitative and qualitative findings	?			?				?	?		?	
	Adherence to quality criteria of methods	✓			X				X	?		✓	
	**Total scores**	**14/17**			**11/17**	**5/7**		**5/7**	**12/17**	**9/17**		**14/17**	**5/7**
	**Percentages**	**82%**			**65%**	**71%**		**71%**	**71%**	**41%**		**82%**	**71%**

* Studies not reporting on realized benefits

√: Yes, X: No, ?: Can't tell
